# Mental health and structural injustice: a qualitative investigation of interlocking patterns of disadvantage among displaced populations in Nairobi, Kenya

**DOI:** 10.1186/s13031-025-00660-6

**Published:** 2025-07-02

**Authors:** D. B. Nieuwe Weme, M. M. Wanga, P. B. Mastaki, J. Osur, W. A. Tol

**Affiliations:** 1 ARQ International, ARQ National Psychotrauma Centre, Diemen, the Netherlands; 2 Health Systems Strengthening directorate, Amref Health Africa, Langata Rd, Nairobi, Kenya; 3 Department of Research, Amref International University, Nairobi, Kenya; 4 Section of Global Health, Department of Public Health, University of Copenhagen, Copenhagen, Denmark; 5 Athena Institute, Vrije Universiteit Amsterdam, Amsterdam, Netherlands

**Keywords:** Refugees, Mental health, Wellbeing, Urban settings, Structural injustice, Intersectionality

## Abstract

**Background:**

Urban refugees face systemic disadvantages that significantly impact their mental health and overall wellbeing. Research on refugee mental health often examines risk factors in isolation, overlooking broader systemic patterns. This study applies a structural injustice framework to investigate how interconnected disadvantages shape the mental health of urban refugees.

**Methods:**

A thirteen-month study examined the wellbeing of Somali and Congolese refugees in Nairobi, employing ethnographic methods, including 69 in-depth interviews, field notes, and observations. Findings were interpreted using Powers and Faden’s structural injustice theory.

**Results:**

Analysis revealed a systemic pattern linking immigration status, sexual and gender-based violence, and limited access to livelihoods. These interconnected factors impact all six elements of wellbeing identified by Powers and Faden and exhibit the theorized characteristics of structural injustice: pervasive, profound, asymmetric, and near-inescapable.

**Discussion:**

The study highlights the structural nature of challenges faced by urban refugees in Nairobi, emphasizing the need to address interconnected systemic barriers. Understanding these patterns is essential for identifying effective interventions and mitigating risks to refugee mental health and overall wellbeing.

**Conclusion:**

The theory as used in this study sheds new light on the interconnected nature of the environment urban refugees live in. This holistic approach to wellbeing provides clarity on vulnerabilities related to specific social groups.

**Supplementary Information:**

The online version contains supplementary material available at 10.1186/s13031-025-00660-6.

## Background

Kenya currently hosts over half a million registered refugees [1]. Most reside in camps in the north, while approximately 100,000 live in urban areas, primarily Nairobi [2]. Increasingly, refugees are drawn to urban centres by economic opportunities [3]. With over 34,000 Somali and almost 24,000 Congolese refugees, they represent some of Nairobi’s largest refugee groups [4].

Refugees in low- and middle-income countries (LMICs) face daily challenges affecting their mental health, such as lack of legal documents which may hinder access to social services [5], as well as language barriers and obstacles to economic integration [6].

Refugees often prefer living in urban centres as they seek economic independence, thus forgoing structured camp services and increasing their exposure to fragmented urban support systems and structural injustice [7].

Urban refugees may encounter additional challenges distinct from life in formal camps or rural areas. Urban living conditions can be harsh due to crowded neighbourhoods with low standards of living [8, 9]. Refugees face barriers accessing healthcare [10], social housing [11], legal systems [12], and financial services [13]. Even though these services seem to be available to most city dwellers, displaced persons experience limited access due to inequitable distribution of access. Xenophobia, discrimination and lack of legal identity documents are suggested to underlie these barriers [6].

A 2019 UK Research and Innovation (UKRI) international research program, through the Global Challenges Research Fund (GCRF), sought to understand the healthcare needs of Somali and Congolese displaced populations in Eastern DRC, Somalia, Kenya, and South Africa. This study builds on that initiative, drawing on qualitative data collected in Nairobi to explore how legal, social, and economic structures shape urban refugee mental health. Specifically, we examine how systemic barriers create patterns of disadvantage that restrict access to essential services and exacerbate vulnerabilities [9].

### Current research on refugee mental health

While mental health and psychosocial wellbeing have gained recognition within global health discussions [14], limited attention has been given to the social determinants of mental health among displaced populations in low- and middle-income countries (LMICs) [15]. The Lancet Commission (2018) highlighted limited qualitative research on socio-ecological determinants of refugee psychological wellbeing as a critical gap [16]. This paucity of data hampers the development of relevant, holistic services that address prevalent mental health issues and at the same time recognise their socially integrated nature.

Existing literature often examines individual adversities in isolation, rarely exploring how they interrelate to form systematic patterns of disadvantage with complex cause-and-effect dynamics [17]. Without understanding this interplay, developing effective interventions is challenging, and unintended outcomes may arise [18].

Most epidemiological studies in LMICs narrowly focus on trauma exposure and PTSD symptoms [19], unlike research in high-income countries extensively exploring post-migration socio-environmental factors [20].

Refugees in fragile contexts face ongoing adversities such as poverty, sexual and gender-based violence (SGBV), and marginalization, which interact with mental health in complex ways [21]. Recent debates in global mental health critique the focus on reducing psychological symptoms without addressing social determinants of mental health [22, 23]. However, there remains a significant gap in understanding how urban refugees in LMIC settings experience structural disadvantage and its effects on mental health. To address this gap, we apply Powers and Faden’s structural injustice theory, which provides a comprehensive framework for understanding systemic patterns of disadvantage in refugee mental health [24, 25].

### Structural injustice theory

Bioethicists Powers and Faden argue that wellbeing is the ultimate moral goal of public health [24, 25]. They use a wellbeing lens to assess the fairness of social structures. In their latest work, they describe structural injustice as stemming from power asymmetries (domination, exploitation, and social exclusion) that create and sustain disadvantages for specific groups.

The concept of social structure—the interplay of institutions, conventions, and cultural expectations—is central to many justice theories, such as the foundational justice theory by John Rawls [26]. According to Powers & Faden, a key task for justice theories is to specify whether these background social and economic conditions should be interpreted as unfair [24].

In this study, we analyse refugee mental health and wellbeing by operationalising Powers and Faden’s theory, focusing on two components: (1) six core elements of wellbeing and (2) four characteristics indicating structural disadvantage.

Powers and Faden define wellbeing through six core elements: (1) (mental) health; (2) knowledge and understanding; (3) personal security; (4) personal attachments; (5) equal respect; and (6) self-determination. Insufficient levels in these elements indicate potential injustice. These elements serve as analytical tools to assess whether wellbeing is achieved and how deficiencies in one may relate to others.

The theory also posits that structural unfairness arises when patterns are asymmetric, near-inescapable, profound, and pervasive. These conditions indicate unjust social arrangements that impact wellbeing. Definitions and examples of these characteristics are summarized in Table 1.

**Table 1:** Characteristics of structural impact. (adapted from powers and Faden, 2019)

Characteristic of structural impact	Definition	Example
Asymmetric Impact	Disproportionate effects on socially situated groups limiting their options.	Low-income groups often live in areas most vulnerable to environmental disasters (e.g., flooding).
Near-inescapable Impact	Groups are trapped by social forces.	Limited social mobility confines individuals to their social class.
Profound Impact	Persistent threats to all wellbeing elements, with significant harm.	Families may face catastrophic consequences from unexpected medical costs.
Pervasive Impact	Institutional practices exert lifetime effects across all aspects of life.	Immigrants are feeling restrictions in all aspects of their life due to their status as ‘second class’ citizen.

### Objectives

Building on Powers and Faden’s structural injustice theory, this study seeks to uncover systemic patterns of disadvantage affecting the mental health and broader wellbeing of Somali and Congolese refugees living in Nairobi. By applying this framework, we examine how legal precarity, economic exclusion, and social discrimination interact to shape mental health outcomes. This study also aims to contribute to the growing body of evidence that informs policy and intervention strategies tailored to urban refugee populations in LMICs, ensuring a more holistic approach to addressing mental health disparities.

## Methodology

### Overall design

This qualitative study analysed data collected between 2022 and 2023 as part of a broader international research project (2019–2024), which aimed to enhance healthcare access for refugees and internally displaced persons, particularly focusing on protracted displacement and gendered challenges. The overarching project involved partners from South Africa, the Democratic Republic of Congo (DRC), Kenya, Somalia, the United Kingdom, and the Netherlands. This analysis specifically utilized data from the fourth work package, originally examining how gender, sexuality, residency, ethnicity, and socio-economic status influence healthcare-seeking behaviors for conditions associated with prolonged displacement, conflict, and gendered violence.

Initial data review revealed intersections of adversities suggesting broader systemic patterns. Consequently, researchers refined the research question to explicitly focus on structural factors affecting mental health. Recognition that participants’ experiences reflected structural disadvantage beyond individual adversity led to the post-hoc application of structural injustice theory for further analysis. Data were drawn from two Nairobi field sites: Eastleigh, hosting many Somali refugees, and Kawangware, home to many Congolese refugees, selected for their unique insights into the distinct challenges faced by urban refugees.

### Sampling method and size

Participants were selected through purposive and snowball sampling. Two groups were included: healthcare providers and refugees.

- **Healthcare Providers**: Included doctors, nurses, religious leaders, counsellors, community health workers, and traditional healers closely interacting with refugees.
- **Refugees**: Identified through clinics and community centres based on Somali or Congolese origin or roles in refugee care. Second-generation refugees were included, balancing gender and age.

Sixty-nine life-history interviews were conducted from May 2022 to June 2023: 19 Congolese refugees, 26 Somali refugees, and 24 healthcare providers (14 Eastleigh, 10 Kawangware). Research assistants (RAs) documented field notes.

### Procedures and ethical reflections

Data collection was carried out by nine RAs and a research coordinator (RC). All except one of the RAs were of Somali or Congolese origin and resided in the studied neighbourhoods. All were Master’s students trained in ethnographic data collection through a week-long workshop led by the RC. Selected RAs also received training in qualitative coding using Taguette, an open-access analysis software. The team included four women and five men. Interviews were conducted in Somali, Congolese Swahili, French, or English, with translations reviewed for accuracy by bilingual researchers.

Ethical approval was obtained from Kenya’s National Commission for Science, Technology and Innovation (NACOSTI), license no. NACOSTI/P/22/15,312. Participants received clear written and verbal explanations of the study purpose, the voluntary nature of participation, and the right to withdraw without penalty. Given sensitive socio-economic conditions and immigration status discussions, participant expectations were managed to minimize social desirability bias [27].

The authors acknowledge potential limitations, including the use of snowball sampling potentially favouring those with stronger community ties or prior healthcare access. Efforts were made to diversify entry points to minimize this bias. Additionally, the diverse professional and socio-cultural backgrounds of the research team introduced complexities in communication and interpretation of data. Nevertheless, these diverse perspectives were managed through structured collaboration, enhancing the depth and richness of the data analysis.

### Analysis

Interviews were transcribed verbatim and analysed using Taguette, an open-access software. The research coordinator (RC) and research assistants (RAs) initially engaged in inductive coding, developing a final codebook comprising nineteen meta-themes, each containing four to twenty-one codes. This codebook was applied systematically across all transcripts.

Following inductive coding, Powers and Faden’s Structural Injustice analytical framework was applied to identify and interpret systemic patterns of disadvantage. A potential limitation lies in the timing of applying structural injustice theory, as data collection and codebook development were nearly complete when this theoretical framework was adopted [28]. While some argue that theory should guide data collection, the post-hoc application was intentionally chosen to minimize initial biases, although we acknowledge earlier theoretical integration might have enhanced analytical rigor [29].

## Results

Here, we present our results through two components of structural injustice theory: the six core elements of wellbeing and the structural (vs. random or incidental) nature of any identified patterns.

### Self-determination

The data suggests that refugee populations in Nairobi are socially immobilized by the challenging social and economic circumstances they face. Bureaucratic and legal barriers prevent people from obtaining essential identity documents. To clarify this, we will briefly describe the current process for obtaining refugee status.

Refugees entering Kenya should report to the nearest government administrative centre, where they will be issued a document from the Department of Refugee Services (DRS) called a ‘manifest’. This document is valid for 30 days and must be extended until a final refugee status (‘mandate’ or ‘Alien Card’) is granted. To receive this mandate, refugees have to go through a Refugee Status Determination (RSD), an interview done by the DRS to determine eligibility for official refugee status in Kenya. Once refugee status is officially approved, one can apply for a working permit which allows refugees to engage in formal labour [30].

Although seemingly straightforward, the process of acquiring a mandate can take up to 10 years, from the moment of registration with DRS. For most refugees, obtaining a working permit is close to impossible. A Congolese refugee who lives in Kenya since 2009 comments on this process: “I myself have been here 15 years and am still looking for a working permit for which I applied three years ago. Those refugees who have received a working permit can be counted on one hand.” For many refugees, this complex process is unclear and causes them to end up in a situation where they are without valid documentation, which leaves them vulnerable to arrests and deportation.

Refugees reported experiencing a precarious life, facing constant threat of deportation and harassment by government officials. Issues surrounding documentation severely restrict movement and limits social and economic freedom. As one male Somali refugee in his late twenties puts it: “There should be creative ways of rebuilding people’s lives. We should be given identification cards for easy movement. As long as we are living in this confined space, there will be no change forever.”

### Personal attachments

Maintaining family, friendships, and romantic relationships outside Nairobi requires disproportionate effort. Traveling is difficult due to financial constraints or fear of arrest due to lack of legal documentation. Without an identity card, one cannot buy a Kenyan SIM card, preventing calls to relatives and access to mobile money, which is essential in Kenya, where 80% of the population relies on mobile payments [31].

Stigma related to being a victim of SGBV or HIV infection severely hinders refugees’ ability to connect with others, or even continue existing relationships. Women experiencing domestic violence or intimate partner violence (IPV) often do not report the violence and instead self-seclude. Interaction with health workers may also be challenging for persons experiencing stigma, illustrated by a health worker’s account.

There was a case we had, where a lady came here with her husband, she was [HIV] positive and he wasn’t. She was taking the medicine discreetly. She told me that in her [Somali] community, getting that sickness is considered a curse. Her husband is insisting they get tested, what should she do? I had to lie to the couple that I don’t have testing kits and that they give me their number so that I contact them when I restock on testing kits.

*- Female community health worker, mid- 50s, Eastleigh*

### Knowledge and Understanding

Many interviewees reported dropping out of school or not continuing their education in Nairobi due to financial constraints. Unfinished schooling limits livelihood opportunities and reduces the ability to make sound social and economic choices. Some interviewees mentioned receiving financial support from NGOs, family members, or other sources to continue their education, but this support was limited.

“Not being able to work or study makes me a refugee. If I could get a job, I will work. If I get an opportunity for education, I will study. I want to live just like other human beings are doing. When you are not a citizen, but a refugee, you live a very limited life.”

*- Somali man, mid-40s, Eastleigh*

Most Congolese refugees are from eastern DRC and speak Congolese Kiswahili, which is similar to Kenyan Swahili, allowing easy communication with locals. Somali refugees, however, struggle to access public services due to language barriers.

### Equal respect

Disrespect was a common theme expressed by interviewees regarding the local population’s attitude towards them. One Somali woman in her late 50s stated that although she would admit to being a refugee, “I don’t know what is wrong with Nairobi residents. They belittle refugees. I don’t understand why.” Similarly, a Congolese woman in her 30s said, “I will not show any Kenyan my refugee status because they will laugh at me and say I have a bad life.” Another refugee described:

In the community, when they know you are a Congolese, that is a problem. Especially me, who has kids, I cannot hide; they will know. They discriminate you, even at the [water] taps. Someone just enters before you and fetches the water, and there’s nothing you will do.

*- Congolese woman, mid-30s, Kawangware*

On the level of service provision, refugees are denied the respect accorded to citizens. A Congolese refugee explained:

Most of the time, refugees are not considered equal to locals, even if we are qualified for the jobs. We are told we are refugees. If we do the same jobs as Kenyans, they are paid more, and we can do nothing about it.

*- Congolese man, mid-20s, Kawangware*

SGBV victimization is tied to unequal gender respect. Within many traditional Somali communities, acts of SGBV are seen as shameful and are dealt with internally via a practice called *Maslaha*. Violence against female refugees is not a rare phenomenon in Nairobi:

They take advantage of refugees who are desperate, who doesn’t have someone to report to, who doesn’t know where to report to, because you are told you don’t have a document, So, you have to stay in, no going out. So, they get all sorts of punishment, they beat them, they burn them (…). And when it comes to GBV, for the Somali cases, it’s complicated. You try solving, you report the case and then there is what we call Maslah. Maslah is a negotiation, the negotiators are family who will solve it. Now the girl remains suffering psychologically.

*- Female community health workers, end 40s, Eastleigh*

This lack of respect for women is compounded by fear of deportation and prevents many from reporting incidents to officials. Lastly, police were also mentioned by a few as being the ones who harassed women as they knew they were vulnerable and would not be able to report them.

### Personal security

Insecurity permeates the daily life of refugees, with a strong gendered dimension. Poverty, economic insecurity, limited social support, and barriers to public services create precarious living conditions. Coping mechanisms such as substance use and economic risks exacerbate the fragile security of refugee households. SGBV, including IPV, female genital mutilation (FGM), rape, and other forms of sexual assault, were frequently mentioned in interviews. An overall sense of insecurity and vulnerability permeates daily life for many refugees in Nairobi.

“Also, about documentation, it is possible that I, as an adult who is working for the family cannot move at night. If you are arrested you will pay the little you have. A Probox [police car] will be stopped next to you and you will be asked to enter, that’s the problem we face. At sunset when the place of work is closed and you pray Maghrib [evening prayers], you have to come home.

*- Somali man, early-40s, Eastleigh*

### Health

Poverty, sanitation issues, SGBV, limited access to healthcare, and stigma were reported as major health concerns. Sickness, in turn, hinders refugees’ ability to pursue limited social and economic opportunities. Financial constraints keep people from accessing care, which in turn might aggravate the medical condition and also keep persons from working and creating income.

“When someone is sick and does not have access to medication or the hospital, worries and stress multiply. You fall sick yourself because you cannot help that person. You cannot help your family, and there is no money for treatment, rent, or livelihood. It is a very difficult matter, and whoever has that experience knows it is not easy. So, it impacts us a lot.”

*- Somali man, mid-40s, Eastleigh*

Mental health was commonly described as intertwined with all elements of wellbeing. The inability to achieve wellbeing leads to stress, negative emotions, and more severe mental health conditions. Stress was frequently mentioned by interviewees, with different causes and manifestations.

One interviewee explained the stress in everyday life:

When you boil water, it evaporates. So, when a person faces a problem, the brain is pressured, and that is what causes instability. A lot of stress, desire for resettlement, and much more that I cannot summarize now. I just say, leave the solution to God. There is no place you can seek meaningful support. When the boiling gets tough, the content evaporates. When there is sickness, rent, and you cannot manage to multi-task, there is nothing you can do.

*- Somali man, mid-40s, Eastleigh*

Self-determination, health and knowledge are central to this quote, illustrating the pervasive impact of stress on several elements of wellbeing. Besides stress, moderate and severe mental health issues are also mentioned by interviewees.

The most common mental health diagnoses are trauma-related disorders, which are understandable because these people are refugees. Then, depression and anxiety are also common, in addition to trauma-related disorders. Some people had genetically inherited conditions, but when they encountered poverty and danger, these conditions triggered. Bipolar, schizophrenia, or other psychiatric issues that were hidden now surface, triggered by the many problems—poverty, domestic violence, and insecurity.

*- Medical Doctor, mid-60s, Eastleigh*

This quote demonstrates how severe, moderate, and trauma-related mental health issues are linked to the social and economic conditions refugees face.

**Fig. 1:**
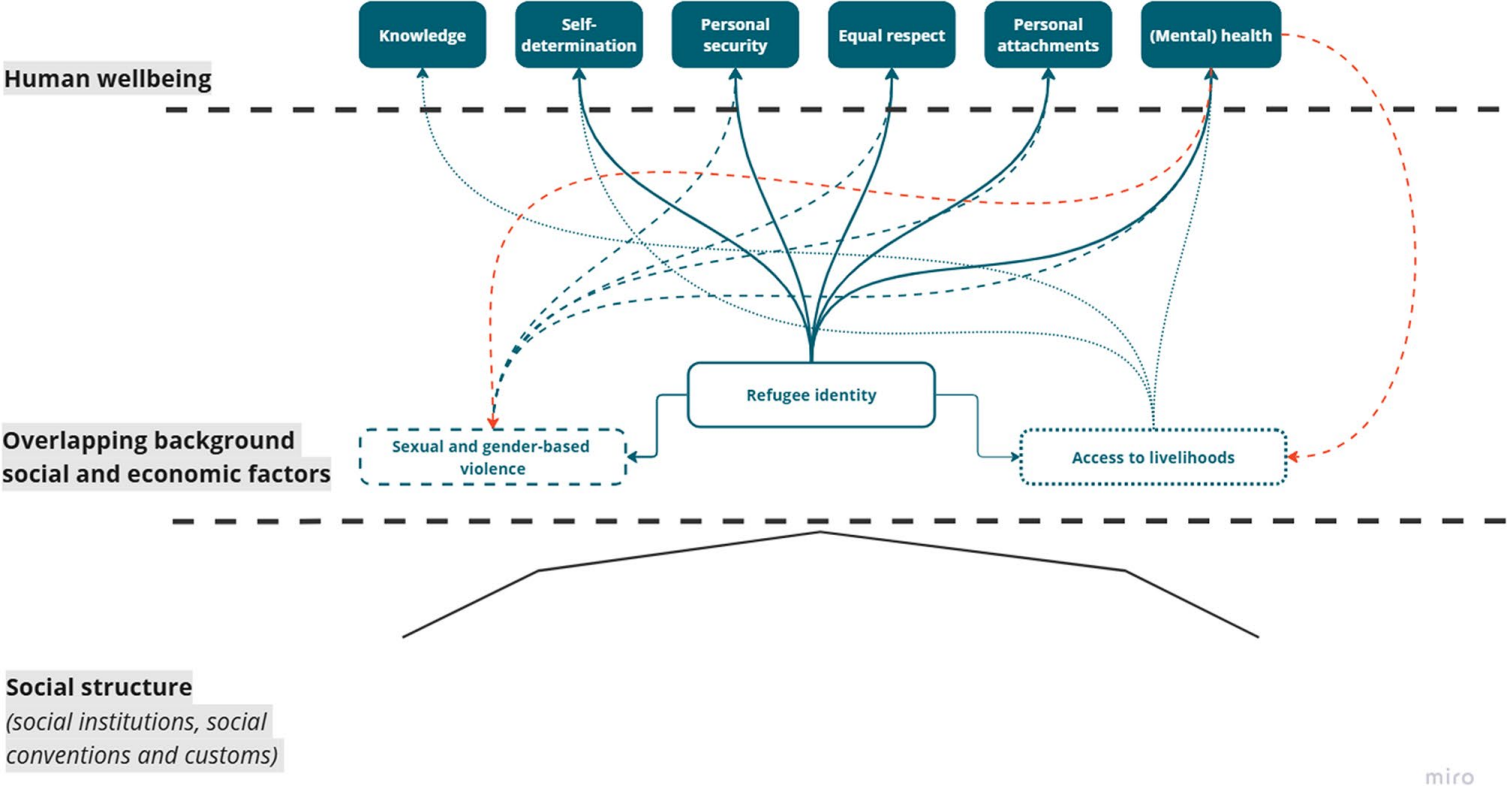
Structural pattern of disadvantage

### What makes impact structural

Social structures are unjust when their effects are asymmetric, near-inescapable, profound, and pervasive. This section explores whether the identified structural pattern of disadvantage (Fig. 1) can be seen as structural according the proposed parameters.

### Asymmetric impact

Refugees in Nairobi find themselves occupying a lower social standing than the host community. Kenyans may have access to healthcare through the National Health Insurance Fund (NHIF), but refugees often lack healthcare access, especially so in urban areas. Participants also reported frequent abuse of power by Kenyan police towards refugees, sometimes leading to imprisonment or demands for bribes. Additionally, refugees face unequal access to both formal and informal job markets. The ‘refugee’ identity closes doors to opportunities before they even arise.

Yes, I see myself as a refugee because a Kenyan born and me, from DRC, because of war, are different. There are things a Kenyan can do but I cannot. There are places a Kenyan can go but I cannot. That is why I consider myself a refugee. There are things a Kenyan kid has the freedom to do but not the case for my kids. I and my kids have no freedom because we are refugees. We live that way and Kenyans call us refugees.

- Congolese woman, early-40s, Kawangware

### Near-inescapable impact

Participants described living in a social system resistant to change, where the influence of social institutions and groups is entrenched in the fabric of society. This creates a resilient system of harm. The effects of this system are shown through restricted social movement and mobility across social classes. A Somali refugee in her mid-30s expressed: “I feel restricted when I see that I cannot be with people and move on with them, since I lack rights.”

### Profound impact

Survival is the default mode for many refugees in Nairobi, making the harmful effects of an unjust social system potentially catastrophic. Refugee populations are severely limited in their social and economic resources, making them vulnerable to deprivation of wellbeing elements. A female Congolese refugee in her mid-30s endured repeated sexual violence. She was fortunate to find work as a housemaid, but the two sons of her employer sexually harassed her. She could not leave the job, as doing so would mean losing income and causing her children to drop out of school. Such ‘choices’ based on systematic harm affect individuals and their families across many aspects of life.

Health issues can result from people not having access to sufficient livelihoods. The problem they now face here on employment, not being allowed to travel outside of Nairobi. You cannot even marry. There is nothing you can do for yourself. There is a lot in which we are restricted, we are in jail that seems no one knows about.

*- Somali male, early-40s, Eastleigh*

### Pervasive impact

The refugee identity, with or without official status, profoundly influences the life of an individual and group. Almost no dimension of life or wellbeing is unaffected by being considered a refugee.

“(…) if you look at livelihoods and mental health, a person is comfortable in their home country. But when you are a refugee in another country with no identity, you are highly controlled. You must be very careful, control yourself, and consider everything.”

*- Somali woman in her 30s, Eastleigh*

## Discussion

Our analysis of 69 interviews over 13 months aimed to identify systemic patterns of disadvantage that affect the mental health and broader wellbeing urban refugee populations through the perspective of structural injustice. Applying structural injustice theory, we found that refugee mental health in Nairobi is shaped by inequality, disadvantage, and discrimination based on refugee status. This finding challenges the idea that single processes alone can improve psychological wellbeing.

By utilising the elements of wellbeing as proposed by Structural Injustice theory we found several social determinants affect refugee wellbeing. Furthermore, interconnections between these determinants were identified, which together coalesce into a pattern of disadvantage. Finally, by analysis of the data through the characters of structural impact, results suggest the impact on refugee wellbeing to be of a highly rigid nature. This resultant pattern will take centre stage in the discussion, in which we will deliberate on the social determinants of mental health and their interconnections.

Three key factors shaped the patterns that collectively affect refugee mental health in Nairobi. These are the perceived and legal identity of a refugee, the access, and the lack of that, to livelihoods, and the risk to, or actual enactment, of acts of SGBV. In the following section these determinants will be discussed in relation to Structural Injustice theory.

### Legal refugee status or identity

Possessing the proper legal documents (and status), as well as perceived identity are found to have pervasive effect on wellbeing. Participants were often ashamed of their refugee status, reflecting low self-respect and oppressive social conditions. These findings align with previous research, suggesting this may increase the risk of mental health problems [32–34]. Caveat here is that most studies have focused on refugees in Europe or the United States.

Additionally, our study found that challenges around legal refugee status and documentation affected the following elements of wellbeing: personal security, equal respect, self-determination, and personal attachments. These findings are less explored in existing literature, with only two known studies applying structural injustice theory to similar issues [35, 36].

The link between legal status and access to livelihoods, two interconnected social factors identified by our study, is well-supported in the literature [3, 37]. Although many countries officially allow refugees to work, most countries have developed complex, tardy bureaucratic processes which forces refugees to work in the informal economy, posing a risk to health and personal security. A 2022 report by the Center for Global Development [38] found major differences when it comes to respecting refugee work rights de jure versus de facto, with half of the assessed countries having a “better environments in law than in practice (including Kenya).”

Jointly, the determinants of livelihoods and legal status affect all six elements of wellbeing, making their impact profound and pervasive.

### Access to livelihoods

Access to work is critical for sustaining life, family, a sense of worthiness and psychological wellbeing. Many interviewees reported challenges in securing livelihoods. Existing research links the lack of vocational opportunities to a felt lack of self-determination [39–41]. Moreover, limited access to livelihoods hinders skill development, particularly so compared to Kenyan nationals.

Participants viewed livelihoods as essential for providing a better future for their children. Literature supports this, highlighting how livelihoods shape refugee identity and relationships between individuals and institutions [42]. Without livelihoods, physical wellbeing is often compromised as insurance is lacking and thus health expenditures are out-of-pocket. Moreover, stress from lack of livelihoods and social connections is strongly linked to mental wellbeing [39, 43].

### Sexual violence

Sexual violence is the third key factor making up the identified pattern. Our study found a potential link between SGBV victimization and lack of documentation, echoing findings from studies on asylum seekers and undocumented migrants in Belgium and the Netherlands [44]. Women in this study felt that that reporting violence was not an option due to the risk of exposing their undocumented status, a barrier also noted in broader refugee research [45]. It could be theorized that legal status is a protective factor as (potential) perpetrators would be more at risk of being reported. Given the sensitivity of this issue, further research is needed to explore this link.

SGBV has long been linked to both physical and mental health issues [46–48]. We argue that SGBV inherently undermines personal security and equal respect, and its impact extends to social relationships and identity [49]. Whereas SGBV typically affects women more than to men, access to livelihoods is applicable to both men and women. Undocumented refugees, men, women and especially children, face daily challenges in attaining wellbeing. Adding either lack of livelihoods or SGBV to refugee status creates a pattern which is nearly-unescapable, pervasive, profound and asymmetric in its effect on wellbeing.

### Future research

It is important to highlight the lack of research on refugee mental health in LMICs, with most studies focusing on HICs despite 73% of displaced people residing in LMICs [50]. This gap may create bias in the literature as well as analysis, and warrants further exploration.

While research on the systemic patterns influencing refugee mental health is growing, much remains unknown [22]. This study contributes to understanding the interconnectedness of underlying social factors and their impact on wellbeing. A systematic review could address these evidence gaps.

Finally, although this study highlights patterns of structural injustice affecting Somali and Congolese refugees, further research should examine the sources of these patterns. This research could focus on processes like racism or patriarchy, specifically on the institutions and mechanisms that perpetuate them. Identifying opportunities for change at both the institutional and social levels is crucial.

### Policy recommendations

Powers and Faden [25] argue that structural solutions are required to address systemic patterns of injustice, emphasizing interdisciplinary approaches. Improving the wellbeing of urban refugees in Nairobi requires an interdisciplinary strategy that acknowledges the interconnected nature of underlying factors. Targeting a single factor is unlikely to yield sustainable results, as other factors continue to affect the same dimensions of wellbeing. Structural, layered interventions designed to enhance refugee wellbeing can provide long-term solutions by addressing the complexity of these interrelated issues.

Insights from multidimensional poverty alleviation strategies recognize poverty’s complexity as a social issue. While monetary poverty remains a significant concern, addressing deprivations in capabilities [51] and intellectual poverty [52] is equally essential to achieving sustainable outcomes.

The global mental health field is similarly moving towards multidisciplinary research and interventions [19]. Our findings—highlighting the impact of refugee status, SGBV, and limited livelihood opportunities on mental health—underscore the necessity for multi-layered, multi-sectoral interventions. In LMICs, where governments often rely on external funding, fostering collaboration among intersectoral working groups is essential. These groups must include refugees, implementing organizations, and other stakeholders such as private sector, to ensure a holistic and systemic approach to supporting mental health. The patterns we identified may serve as a technical guide for these efforts.

However, interdisciplinary collaboration is often hindered by epistemological differences regarding concepts and theories, as well as challenges in communication and resource allocation [53]. Addressing these barriers is critical for fostering effective partnerships. Moreover, technical challenges persist within the mental health field, where the current mental health and psychosocial support (MHPSS) framework emphasizes segregated layers over integrated interventions [54]. Without multi-layered, multi-sectoral collaboration, the full potential of interventions remains unrealized, and unintended consequences may arise [18].

Boundary objects, such as structural injustice theory, offer a promising avenue for uniting multidisciplinary thinking. These objects are deliberately designed to function across disciplines, resource levels, and institutional boundaries [22]. By experimentally applying structural injustice theory as an analytical framework, its utility as a boundary object could be tested. This approach could standardize multidisciplinary practices, support the development of interdisciplinary indicators, and serve as a foundational framework for knowledge creation processes [55].

## Conclusion

Without a full understanding of integrated, social processes that lead to vulnerability and ultimately to disadvantage, addressing wellbeing becomes a futile exercise. The siloed approach among developmental and humanitarian stakeholders hampers sustainable programming impact, as these patterns transcend sectoral boundaries. An increased integrated way of working between disciplines could lead to a more holistic, realistic approach to supporting mental and psychosocial wellbeing.

Governmental bodies and social conventions are rigid and resistant to change, and yet these are the very structures in which injustice is ingrained. Agreed that it does take time to address such structures and resulting processes, knowledge on how underlying processes create disadvantage can lead to sustainable methods of improving wellbeing. The sustainability sits within the fact that separate interventions can be improved upon and connected, creating systems which make sense to both those who deliver and receive.

## Electronic supplementary material

Below is the link to the electronic supplementary material.

Supplementary Material 1

## Data Availability

All data from the GCRF funded DiSoCo project is currently being cleaned and will be uploaded to the UK Data Service per policy of the GCRF. Data are available from the corresponding author on reasonable request.

## Abbreviations

DRC: the Democratic Republic of Congo
FGM: female genital mutilation
HIV: Human Immunodeficiency Virus
IPV: Intimate Partner Violence
LMICs: Low- and Middle-Income Countries
MHPSS: Mental health and psychosocial support
NGOs: Non-governmental organisations
NHIF: National Health Insurance Fund
RAs: Research assistants
RC: Research coordinator
SGBV: Sexual and gender-based violence
