# Supplementary figures and images for: Mental health and structural injustice: a qualitative investigation of interlocking patterns of disadvantage among displaced populations in Nairobi, Kenya
Source: Confl Health. 2025 Jul 2;19:40. doi: 10.1186/s13031-025-00660-6 (PMC12224868; doi:10.1186/s13031-025-00660-6)

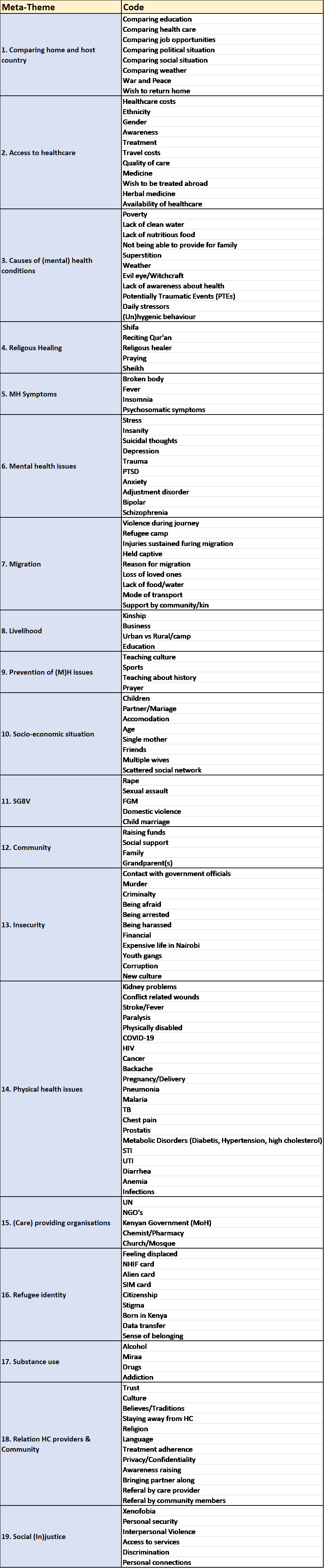

Supplement: Supplementary file 1 — Supplementary Material 1 [file 13031_2025_660_MOESM1_ESM.png]
